# Radiotherapy-Induced Weight Loss and Malnutrition in Head and Neck Cancer: A Prospective Observational Study

**DOI:** 10.7759/cureus.111690

**Published:** 2026-06-28

**Authors:** Shebin George, Simran Kaur, Abraham P Abraham, Ashish Varghese, Shekhar Kapoor, Preeti Negi, Pamela A Kingsley

**Affiliations:** 1 Medical Oncology, Smita Memorial Hospital and Research Center, Thodupuzha, IND; 2 Radiation Oncology, Christian Medical College Ludhiana, Ludhiana, IND; 3 Otolaryngology - Head and Neck Surgery, Medical City for Military and Security Services Hospital, Muscat, OMN; 4 Oral Medicine and Radiology, Christian Dental College Ludhiana, Ludhiana, IND; 5 Radiation Oncology, Capitol Hospital, Jalandhar, IND

**Keywords:** cancer of the head and neck, malnutrition, pg-sga, radiotherapy (rt), support care, weight loss

## Abstract

Background

Head and neck cancers represent a significant global health burden, with radiotherapy being a cornerstone treatment modality. However, treatment-related nutritional complications significantly impact patient outcomes and quality of life. This study aimed to evaluate the impact of radiation therapy on nutritional status and explore the relationship between treatment-induced weight loss and clinical outcomes in head and neck cancer patients undergoing definitive or adjuvant radiotherapy.

Methodology

A prospective observational study was conducted over 18 months (September 2015 to March 2017) involving 83 patients with histopathologically confirmed head and neck squamous cell carcinoma receiving radiotherapy or chemoradiotherapy at a tertiary care center. Nutritional assessment was performed using anthropometric measurements and Patient-Generated Subjective Global Assessment (PG-SGA) at baseline, week three, treatment completion, and six weeks post-treatment. Statistical analysis was performed using SPSS version 21.0.

Results

The study population had a mean age of 57 years with a male predominance (81.93%). The oral cavity was the most common primary site (43.37%). Baseline malnutrition prevalence was 78.31%, increasing to 100% during treatment. Critical weight loss (>5%) occurred in 90.36% of patients, with significant site-specific variations (p = 0.029). Universal weight loss was observed in larynx/hypopharynx and oropharynx patients compared to 82% in oral cavity cancers. Treatment volume correlated significantly with weight loss at weeks 5, 6, and 12 (p < 0.05).

Conclusions

Radiotherapy for head and neck cancer results in universal malnutrition with significant anatomical site-specific variations. Proactive nutritional intervention strategies are essential, particularly for patients with larynx/hypopharynx and oropharynx primaries.

## Introduction

Head and neck cancers are classified by the anatomical area from which they arise in the oral cavity, comprising the lips, anterior two-thirds of the tongue, hard palate, buccal mucosa, gums, floor of the mouth and nasopharynx, oropharynx, larynx, nasal cavity and paranasal sinuses, and salivary glands [1]. According to the Global Cancer Observatory 2022 estimates, the incidence of head and neck cancers in India was 233,298 cases [2]. The etiology of head and neck cancers is multifactorial, with significant regional variations in risk factor prevalence. In India, lifestyle factors, including smokeless tobacco use and areca nut consumption, are primary causative factors [3], while Western populations demonstrate higher associations with cigarette smoking and alcohol abuse. Additional risk factors include viral infections (human papillomavirus predisposing to oropharyngeal cancers and Epstein-Barr virus to nasopharyngeal carcinomas), poor nutritional status, genetic susceptibility, and occupational exposures [4].

Current treatment paradigms emphasize multidisciplinary care involving otolaryngologists, medical oncologists, radiation oncologists, and allied health professionals [5]. Surgery and/or radiotherapy with various chemotherapy regimens form the standard treatment approach [6]. Concurrent chemoradiotherapy provides an absolute five-year survival benefit of 8% compared to radiotherapy alone, achieving superior local and organ preservation in locally advanced cases [7]. However, radiotherapy induces significant treatment-related toxicities, including dysphagia, odynophagia, oral mucositis, xerostomia, dysgeusia, and fatigue, which are accentuated by concurrent chemotherapy [8]. These complications, particularly mucositis-induced dysphagia, represent the most prominent treatment-related toxicity, leading to increased hospitalization rates and treatment interruptions [9].

Malnutrition is a critical concern in head and neck cancer patients, with some studies reporting approximately 49% of patients being malnourished at treatment initiation. This prevalence increases to 71% by the end of treatment, likely due to treatment-related side effects [10]. The nutritional sequelae may persist for several weeks following treatment completion and may have prognostic significance [11]. Some studies have further shown that regular monitoring of anthropometric parameters can serve as a useful surveillance method in head and neck cancer management [12]. Hence, this study aimed to comprehensively evaluate the nutritional impact of radiotherapy in head and neck cancer patients and identify predictive factors for treatment-related nutritional decline.

## Materials and methods

Study design and participants

This prospective observational study was conducted at the Department of Radiotherapy, Christian Medical College, Ludhiana, over 18 months (September 2015 to March 2017). The study protocol was approved by the Institutional Ethics Committee (approval number: cmc/4532), and written informed consent was obtained from all participants. Inclusion criteria were all patients with histopathologically confirmed head and neck squamous cell carcinoma who were scheduled for definitive or adjuvant radiotherapy/chemoradiotherapy, aged ≥18 years, with an Eastern Cooperative Oncology Group (ECOG) performance status of 0-3. All patients with skin or ear malignancies, previous radiotherapy to the head and neck region (re-irradiation cases), presence of distant metastasis, any significant cardiac or hepatic failure, and those unable to provide informed consent were excluded from the study following screening. The sample size was calculated with 80% power and a significance level of 0.05, resulting in the enrollment of 83 patients. External beam radiotherapy was delivered using an Elekta Compact 6 MV linear accelerator employing three-dimensional conformal radiotherapy or intensity-modulated radiotherapy techniques as follows: Initial phase: 40 Gy in 20 fractions over four weeks; boost phase 1: 20 Gy in 10 fractions over two weeks; boost phase 2: 6-10 Gy in 3-5 fractions; total dose: 66-70 Gy for residual gross tumor. Concurrent chemotherapy regimens were administered as per institutional protocols for patients receiving chemoradiotherapy.

Assessments

Anthropometric measurements, including height, weight, body surface area, and body mass index (BMI), were documented at baseline and weekly throughout treatment. Weight measurements were performed using calibrated digital scales. Patient-Generated Subjective Global Assessment (PG-SGA) was used. Nutritional status was assessed at baseline, week three, treatment completion, and six weeks post-treatment. PG-SGA grades were categorized as follows: A (well-nourished), B (moderately malnourished), and C (severely malnourished). Malnutrition criteria were unintentional weight loss >10% over any time period, weight loss >5% over three months, combined with BMI <18.5 kg/m². Critical weight loss was defined as >5% from the baseline. Hemoglobin and serum albumin levels were monitored weekly during treatment. Acute toxicities were graded according to Common Terminology Criteria for Adverse Events version 4.0. Xerostomia was graded as 0-3 based on severity, and mucositis was graded as 0-3 based on clinical assessment.

Statistical analysis

Data analysis was performed using SPSS version 21.0 (IBM Corp., Armonk, NY, USA). Descriptive statistics were used for demographic and clinical characteristics. Paired t-tests were employed for continuous variables, and chi-square tests for categorical variables. Correlation analysis was performed to assess relationships between treatment volume and weight loss patterns. Statistical significance was set at a p-value <0.05.

## Results

The mean age of the study population was 57 years (range = 26-93 years). Most patients (59.03%) belonged to the 46-65-year age group, and the majority were males (81.93%). Overall, 77.1% of patients had an ECOG performance status of 0-1 (Table 1).

**Table 1:** Patient demographics and baseline characteristics (N = 83). ECOG = Eastern Cooperative Oncology Group

Parameter	Category	Number of patients	Percentage (%)
Age distribution	26–35 years	3	3.61
	36–45 years	14	16.87
	46–55 years	21	25.30
	56–65 years	28	33.73
	66–75 years	10	12.05
	>75 years	7	8.43
Gender	Male	68	81.93
	Female	15	18.07
ECOG performance status	0	3	3.61
	1	61	73.49
	2	16	19.28
	3	3	3.61

The primary site of involvement was the oral cavity and oropharynx in 61.46% of the study population. Overall, 67.47% of patients had locally advanced disease (stage III-IV). Definitive concurrent chemoradiation was the most common treatment modality offered (61.45%) (Table 2).

**Table 2:** Disease characteristics and treatment modalities.

Parameter	Category	Number of patients	Percentage (%)
Primary site	Oral cavity	36	43.37
	Larynx	20	24.09
	Oropharynx	15	18.09
	Hypopharynx	4	4.81
	Nasopharynx	3	3.61
	Maxilla	3	3.61
	Nasal cavity	2	2.41
Disease stage	Stage I	10	12.05
	Stage II	17	20.48
	Stage III	20	24.10
	Stage IV	36	43.37
Treatment modality	Radical radiotherapy	17	20.48
	Chemoradiotherapy	51	61.45
	Adjuvant radiotherapy	4	4.82
	Adjuvant chemoradiotherapy	11	13.25

At baseline, according to the BMI criteria for the Asian population, 16.87% of our patients were undernourished, and 78.31% of patients were malnourished as per PG-SGA, with 45 (54.22%) patients reporting PG-SGA grade B nutritional status (Table 3).

**Table 3:** Nutritional status assessment at baseline. BMI = body mass index; PG-SGA = Patient-Generated Subjective Global Assessment

Parameter	Category	Number of patients	Percentage (%)
BMI classification	Underweight (<18.5 kg/m²)	14	16.87
	Normal (18.5–24.9 kg/m²)	48	57.83
	Pre-obese (25–29.9 kg/m²)	13	15.66
	Class I obesity (30–34.9 kg/m²)	8	9.64
PG-SGA grade	A (well-nourished)	18	21.69
	B (moderate malnutrition)	45	54.22
	C (severe malnutrition)	20	24.10
Overall malnutrition	Present (PG-SGA B/C)	65	78.31
	Absent (PG-SGA A)	18	21.69

The mean BMI at treatment initiation was 23 kg/m², which decreased to a minimum at the sixth week (20.94 kg/m²). The BMI showed a minimal rise at the 12th week (21.52 kg/m²) but did not return to the baseline. The change in BMI during treatment compared to the baseline BMI was statistically significant from the second week to the 12th week (p < 0.0001) (Table 4). Overall, 90.36% of the study population had critical weight loss, either during treatment or during follow-up by the 12th week.

**Table 4:** BMI changes during treatment course. BMI = body mass index

Time point	Mean BMI (kg/m²)	Standard deviation	P-value (vs. baseline)
Baseline	23.00	±1.85	-
Week 1	22.86	±1.82	0.051
Week 2	22.49	±1.78	<0.0001
Week 3	22.04	±1.74	<0.0001
Week 4	21.71	±1.70	<0.0001
Week 5	21.44	±1.67	<0.0001
Week 6	20.94	±1.63	<0.0001
Week 12	21.52	±1.69	<0.0001

**Figure 1:**
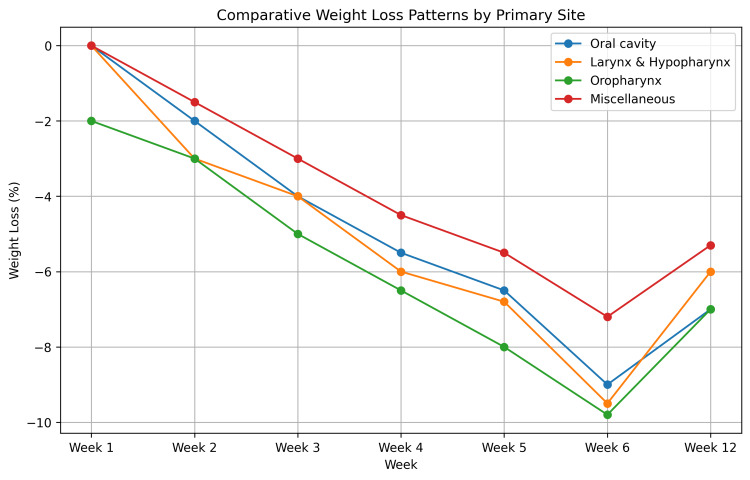
Weight loss patterns during treatment by the primary site.

Patients with nasal cavity and maxilla primaries had the least change in weight during treatment compared to other primary sites (Figure 1).

The association of critical weight with the primary site was found to be statistically significant (p = 0.029). All patients (100%) with the primary site in the larynx/hypopharynx and oropharynx had critical weight loss. In patients with the primary site in the oral cavity, 82.35% had critical weight loss (Table 5).

**Table 5:** Critical weight loss analysis.

Parameter	Category	Number of patients	Percentage (%)	P-value
Overall critical weight loss	Present	75	90.36	-
	Absent	8	9.64	
By primary site				0.029
Oral cavity	Critical weight loss	28	82.35	
	No critical weight loss	6	17.65	
Larynx and hypopharynx	Critical weight loss	24	100.00	
	No critical weight loss	0	0.00	
Oropharynx	Critical weight loss	17	100.00	
	No critical weight loss	0	0.00	
Miscellaneous	Critical weight loss	6	75.00	

By the third week, the nutritional status of patients deteriorated, with 54 (65.06%) patients developing grade C nutritional status, which further increased to 74 (89.16%) patients at the sixth week. By the 12th week, patients reported an improvement in nutritional status, with 74 (89.16%) patients attaining grade B nutritional status (Table 6).

**Table 6:** PG-SGA grade distribution during treatment.

Time point	Grade A, n (%)	Grade B, n (%)	Grade C, n (%)	P-value
Baseline	18 (21.69)	45 (54.22)	20 (24.10)	-
Week 3	1 (1.20)	28 (33.73)	54 (65.06)	<0.0001
Week 6	0 (0.00)	9 (10.84)	74 (89.16)	<0.0001
Week 12	7 (8.43)	74 (89.16)	2 (2.41)	<0.0001

The prevalence of malnutrition was assessed by BMI, PG-SGA, and weight loss >10%. The prevalence of malnutrition at baseline according to BMI was 16.87%, which increased to 26.51% by six weeks. Based on PG-SGA grading, the prevalence increased from 78.31% at baseline to 100% by six weeks. Based on patients with weight loss >10%, the prevalence of malnutrition was 36.14% at six weeks. All patients with PG-SGA grade B/C were found to have a BMI <18.5 kg/m².

## Discussion

Cancer continues to rank among the top causes of death globally and represents a significant challenge to enhancing life expectancy and quality of life [13]. This prospective study demonstrates the profound impact of radiotherapy-induced weight loss on head and neck cancer patients, with universal progression to malnutrition during treatment and significant implications for survival outcomes. Our findings provide critical insights into the relationship between treatment-related nutritional decline and clinical outcomes in this vulnerable population.

The demographic characteristics of our study population align well with established epidemiological patterns for head and neck cancers, with most patients presenting between the ages of 26 and 93 years [14]. However, our study population demonstrated relatively poorer performance status compared to other international studies (77.1% vs. ~85% ECOG 0-1) [15,16], which may reflect delayed diagnosis and late stage at presentation to oncological care, a pattern commonly observed in resource-limited healthcare settings with direct implications for baseline nutritional status and subsequent survival outcomes.

The finding that 90.36% of patients experienced critical weight loss (>5%) with a significant association with the primary site (p = 0.029) has profound implications for survival. The universal occurrence of critical weight loss in larynx/hypopharynx and oropharynx primaries compared to 82% in oral cavity cancers reflects the anatomical challenges these sites present for maintaining adequate nutritional intake during treatment [17]. This site-specific vulnerability is particularly concerning given that weight loss >5% during radiotherapy has been consistently associated with poorer survival outcomes in multiple studies. The functional implications of treating larynx/hypopharynx and oropharyngeal regions, sites critical for swallowing function, result in radiation-induced edema, mucositis, and fibrosis that significantly compromise nutritional intake and, consequently, treatment tolerance and survival. The high baseline malnutrition prevalence (78.31%; PG-SGA grades B/C) compared to the study by Wallmander et al. [10] (49%) highlights the nutritional vulnerability of our population with poorer treatment outcomes. The universal progression to malnutrition during treatment (100% by week six) represents a critical finding, as severe malnutrition has been consistently associated with increased treatment-related complications, higher rates of treatment interruptions, prolonged hospitalization, compromised immune function, reduced treatment tolerance, and poorer overall survival outcomes. The PG-SGA tool demonstrated limitations in our setting, with poor sensitivity (5%) for detecting acute nutritional changes. This limitation is particularly concerning from a survival perspective, as delayed recognition of nutritional decline may result in missed opportunities for interventions that could impact treatment outcomes and survival.

The significant BMI decline from 23.0 kg/m² to 20.94 kg/m² during treatment, with only modest recovery to 21.52 kg/m² at 12 weeks, demonstrates the persistent impact of treatment on nutritional status. Unlike Arribas et al. [18], who observed continued decline post-treatment, our patients showed improvement, suggesting effective nutritional support strategies that may positively impact survival outcomes. The incomplete recovery of BMI by 12 weeks post-treatment raises concerns about long-term nutritional status and its impact on survival. Patients who fail to recover adequate nutritional status following treatment completion may face increased susceptibility to infection, delayed wound healing, and compromised quality of life, all factors that can indirectly affect survival outcomes.

Limitations

This study has several limitations that should be considered while interpreting the findings. Being a single-center study with a relatively small sample size (n = 83), the generalization of the results to wider and more diverse populations is limited. The observational design precludes establishing a definitive causal relationship between radiotherapy-induced weight loss and survival outcomes. Additionally, the follow-up period was restricted to 12 weeks post-treatment, which limits the assessment of long-term nutritional recovery, late toxicities, and their impact on oncological outcomes such as overall and disease-free survival. The study cohort included patients receiving both radiotherapy alone and concurrent chemoradiotherapy, introducing treatment heterogeneity that may have influenced the degree of nutritional decline. Furthermore, although nutritional assessment was performed using PG-SGA and anthropometric parameters, more objective and sensitive measures, such as body composition analysis and detailed dietary assessments, were not incorporated. Potential confounding factors, including socioeconomic status, comorbidities, and variability in nutritional support, were also not fully accounted for.

Future directions

Future research should focus on large, multi-center prospective studies with extended follow-up to better define the long-term impact of treatment-related weight loss on survival outcomes and quality of life in head and neck cancer patients. Integration of advanced and objective nutritional assessment tools, including sarcopenia evaluation and metabolic markers, may allow earlier identification of at-risk patients. There is a need for randomized controlled trials to evaluate the efficacy of proactive nutritional interventions such as early enteral feeding, individualized dietary counseling, and multidisciplinary supportive care in improving treatment tolerance and clinical outcomes. Development of predictive models incorporating tumor site, treatment factors, and baseline nutritional status could facilitate risk stratification and personalized care. Additionally, site-specific nutritional management strategies, particularly for high-risk groups such as oropharyngeal and hypopharyngeal cancers, should be explored. Addressing broader health system challenges, including delayed presentation and limited access to nutritional support, may further contribute to improved patient outcomes in resource-constrained settings.

## Conclusions

This study demonstrates that radiotherapy for head and neck cancer is associated with a near-universal progression to malnutrition, with significant variation by anatomical site, particularly in the oropharynx and hypopharynx. The observed association between nutritional decline and potential survival compromise underscores malnutrition not merely as a treatment side effect but as a modifiable prognostic factor. The high prevalence of baseline malnutrition (78.31%) and critical weight loss (90.36%) underscores the need for early, site-specific nutritional assessment and intervention. Tumor location emerged as a key predictor of nutritional decline due to treatment-related toxicities such as mucositis, edema, and fibrosis, all of which severely impair oral intake. These findings highlight malnutrition as a modifiable prognostic factor that may adversely impact treatment tolerance, quality of life, and survival outcomes, emphasizing its integration into routine cancer care, especially in resource-limited settings.
